# Cryptotanshinone inhibits human glioma cell proliferation *in vitro* and *in vivo* through SHP-2-dependent inhibition of STAT3 activation

**DOI:** 10.1038/cddis.2017.174

**Published:** 2017-05-11

**Authors:** Liang Lu, Sulin Zhang, Cuixian Li, Chun Zhou, Dong Li, Peiqing Liu, Min Huang, Xiaoyan Shen

**Affiliations:** 1 Department of Pharmacology, College of Pharmacy, Guilin Medical University, Guilin, Guangxi, China; 2 Department of Pharmacology and Toxicology, School of Pharmaceutical Sciences, Sun Yat-Sen University, Guangzhou, Guangdong, China; 3 Department of Pharmacology, School of Pharmacy, Fudan University, Shanghai, China; 4 Department of Physiology, School of Basic Medical Sciences, Southern Medical University, Guangzhou, Guangdong, China; 5 Medical Immunopharmacology Research Center, School of Pharmaceutical Sciences, Southern Medical University, Guangzhou, Guangdong, China

## Abstract

Malignant gliomas (MGs) are one of the most common primary brain cancers in adults with a high mortality rate and relapse rate. Thus, finding better effective approaches to treat MGs has become very urgent. Here, we studied the effects of cryptotanshinone (CTS) on MGs *in vitro* and *in vivo*, and explored the underlying mechanisms. Effects of CTS *in vitro* on cell proliferation, cycle, migration and invasion were evaluated. The activation of JAK/STATs signaling was detected by western blot and immunofluorescenc staining. SHP-2 inhibitor or SiRNA were used to determine the involvement of SHP-2. The *in vivo* anti-MGs activity of CTS was studied with nude mice bearing intracerebral U87 xenografts. Our results revealed that CTS significantly inhibited the proliferation of MGs *in vitro* via inhibiting STAT3 signal pathway. The cell cycle was arrested at G0/G1 phase. Although CTS did not change the expression of total SHP-2 protein, the tyrosine phosphatase activity of SHP-2 protein was increased by CTS treatment in a dose-dependent manner *in vivo* and *in vitro*. SHP-2 inhibitor or SiRNA could reverse the inhibitory effect of CTS on phosphorylation of STAT3 Tyr705. *In vivo* study also showed that CTS inhibited the intracranial tumor growth and extended survival of nude mice bearing intracerebral U87 xenografts, confirming an inhibitory effect of CTS on MGs. Our results indicated CTS may be a potential therapeutic agent for MGs. The inhibitory action of CTS is largely attributed to the inhibition of STAT3 Tyr705 phosphorylation with a novel mechanism of upregulating the tyrosine phosphatase activity of SHP-2 protein.

Signal transducer and activator of transcription 3 (STAT3) belongs to the STATs family, and may be the most intimately linked to tumorigenesis.^1^ Activation of STAT3 is initiated by phosphorylation, followed by dimerization and translocation into the nucleus to regulate transcription of target genes.^2^ As a master transcription factor and also an oncogene protein, STAT3 is transiently activated in normal cell, but is constitutively activated in malignant gliomas (MGs). In 2010, a literature published in *Nature* reported that STAT3 has an important role in mesenchyme differentiation and predicts poor clinical outcome in human glioma.^3^ Thus, STAT3 signaling is considered a potential target for therapeutic intervention of MGs.^4, 5^ Many STAT3 modulators have been demonstrated, such as metal complex,^6, 7^ peptide,^8^ small interference of specific RNA (SiRNA),^9^ natural products.^10^ STAT3 activation is negatively regulated by protein tyrosine phosphatase (PTPs), protein inhibitor of activated STAT family proteins (PIAS), suppressor of cytokine signaling (SOCS) and ubiquitin–proteasome pathway.^11^ For instance, some PTPs, such as SHP-1, SHP-2 and TC-PTP, can regulate STAT3 activation through tyrosine dephosphorylation.^12, 13, 14^ Activating SHP-1 by Morin was reported to have an inhibitory effect on STAT3 activation.^15^

Using structure-based virtual screening method, a lot of STAT3 inhibitors from nature products have been identified.^16, 17^ Cryptotanshinone (CTS), one of the major representative components isolated from the root of Salvia miltiorrhiza Bunge has been reported to have inhibitory effects on STAT3 activation. It was also reported to have other pharmacological effects including anticardiovascular diseases,^18^ anti-inflammation,^19, 20^ neuron protection^21^ and antiproliferation of cancer cells.^22^ We recently observed that CTS suppressed the proliferation of human MG cells T98G and U87 through inhibition of STAT3 signaling pathway.^23^ However, little is known about its *in vivo* effect on tumor, particularly MGs. Because CTS can pass through the blood–brain barrier,^24^ we inferred that CTS may have a potential therapeutic effect for MGs. In the present study, we explored the role and mechanism of CTS by both *in vitro* and *in vivo* MG models. Our results disclose that CTS may be a potential drug candidate for the treatment of MG via upregulating the PTP activity of SHP-2.

## Results

### CTS had an inhibitory effect on the proliferation of human MG cells

**Figure 1:**
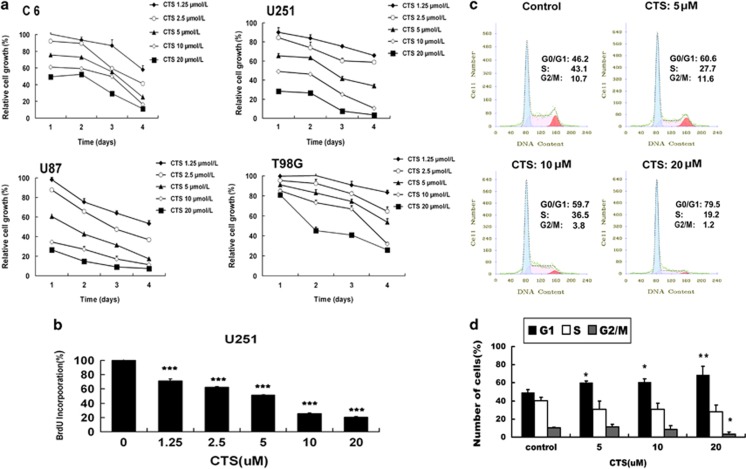
Effects of CTS on the proliferation and cell cycle of glioma cell lines. (**a**) C6, U251, T98G or U87 cells were treated with different doses of CTS (0–20 *μ*M) for the indicated time. Cell growth was measured by MTT assay. Data are expressed as mean±S.E.M.; *n*=3 for each group. (**b**) The proliferation of U251, T98G or U87 cells treated with different doses of CTS for 48 h was measured by BrdU cell proliferation assay. Data are expressed as mean±S.E.M.; *n*=3 for each group. ***P<0.001 *versus* control group. (**c**) Representative flow cytometry images of cell cycle analysis of U251 cells treated with indicated concentrations for 24 h. (**d**) The G0/G1, S and G2/M fractions were measured. Data are expressed as mean±S.E.M.; *n*=3 for each group. *P<0.05; **P<0.01 *versus* control group

To explore the effects of CTS on the growth of MG cells, four MG cell lines, C6, U251, U87 and T98G were treated with CTS at different concentrations (0, 1.25, 2.5, 5, 10, 20 *μ*M) for different time periods (1, 2, 3, 4 days). The results showed that the proliferation of C6, U251, U87 and T98G cells was all inhibited by CTS in a time- and dose-dependent manner (Figure 1a). According to the cell growth curves, the half maximal inhibitory concentration (IC50) of CTS was calculated. For C6, U251, U87 and T98G cells, the IC50 value was 18.09, 7.63, 3.94 and 13.17 *μ*M, respectively for 2 days treatment. Especially, CTS exhibited the most remarkable growth inhibitory effect on U87 cells. To further confirm the inhibitory effect of CTS, U251 cells were treated with different doses (0, 1.25, 0.25, 5, 10, 20 *μ*M) of CTS for 48 h followed by BrdU cell proliferation ELISA kit measurement (Figure 1b). The results showed that the proliferation of U251 cells was inhibited by CTS in a dose-dependent manner. This result was consistent with the results from MTT assay. To explore the effect of CTS on human MG cell cycles, U251 cells were treated with CTS for 24 h followed by cell cycle analysis. As shown in Figure 1c, CTS at the dose from 5 to 20 *μ*M arrested cell cycle at G0/G1 phase, the percentage of cells in G0/G1 phase raised from 49.2 to 68.1%. Accordingly, the percentage of cells in G2/M phase decreased from 10.3 to 3.4% (Figures 1c and d). These results indicated that CTS might be one kind of antiproliferative drug, which has great effect on cell cycle arrest in MG cells.

### CTS inhibited migration and invasion of U251 cells

**Figure 2:**
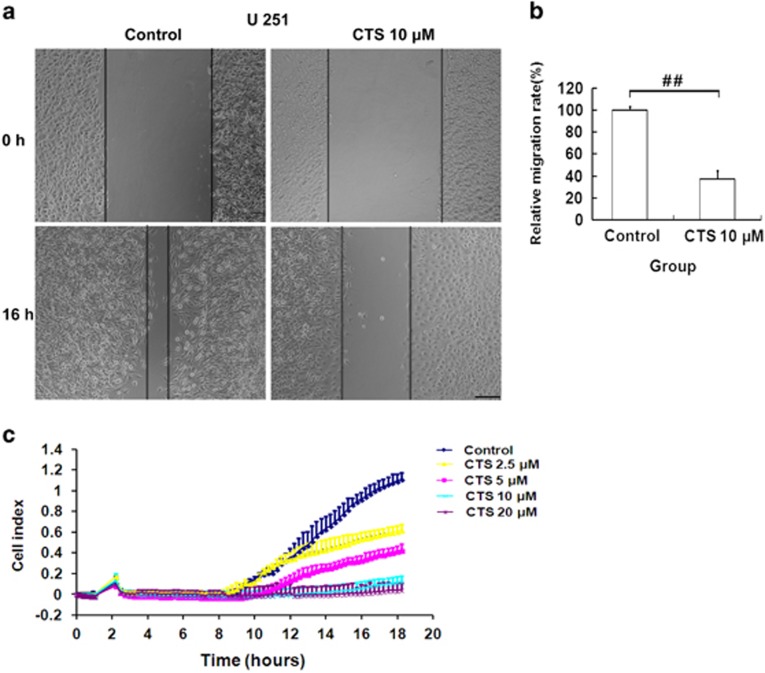
Effects of CTS on cell migration and invasion of U251 cells. (**a**) Migration was evaluated using scratch wound assay. A scratch was made in the confluent monolayer. Representative images are shown at 0 h and after 16 h treatment with or without CTS treatment. Scale bar, 200 *μ*m. (**b**) Bars represent the relative migration index of CTS treatment, expressed as a value relative to the distance moved by the cell monolayer compared with control group. Results were expressed as mean±S.E.M. (*n*=3 for each group). ^##^*P*<0.01 *versus* control group. (**c**) Invasion was evaluated using the xCELLigence System. Representative cell invasion kinetics analysis of U251 cells treated with indicated concentrations CTS for 18 h. Results are expressed as mean±S.E.M. (*n*=3 for each group)

Next, we examined the influence of CTS on migratory capacity of U251 cells using scratch wound assay. After injury of tipping scratch, the U251 cells were treated with CTS (10 *μ*M) for 16 h. Cell migration into the wound was measured according to the distance between the wound edges before and after CTS treatment. We observed that CTS significantly impaired cell migration into the wound (Figure 2a), and cell migration distance was decreased almost 60% compared with the control group (Figure 2b). Besides, cell invasion was evaluated using the xCELLigence system. As shown in Figure 2c, the invasion ability of U251 cells was inhibited by CTS in a dose-dependent manner, and 20 *μ*M CTS treatment almost completely inhibited invasion of U251.

### Inhibitory effect of CTS on glioma cell proliferation is dependent on the suppression of STAT3 activation

**Figure 3:**
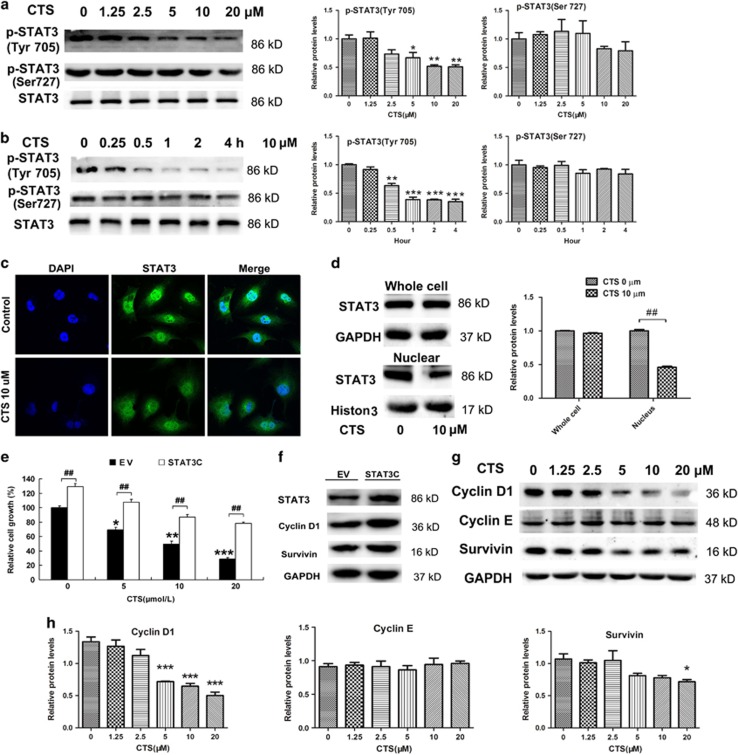
Inhibitory effect of CTS on glioma cell proliferation is dependent on the suppression of persistent STAT3 activation. (**a**) U251 cells were treated with the indicated concentration of CTS for 2 h. The expression of proteins was analyzed by western blotting with indicated antibodies. The bands were then quantified by ImageJ software. Results are the mean±S.E.M. (*n*=3 for each group). *P<0.05, **P<0.01 *versus* control. (**b**) U251 cells were treated with 10 *μ*M CTS for the indicated time, followed by western blot with the indicated antibodies. The bands were then quantified by ImageJ software. Results are the mean±S.E.M. (*n*=3 for each group). ***P<0.001 *versus* control. (**c**) U251 cells were treated with CTS (10 *μ*M) for 120 min. Representative confocal immunofluorescent images revealed the nuclear levels of STAT3 (green). (**d**) U251 cells were treated with CTS (10 *μ*M) for 120 min, followed by nuclear protein isolation. The expression levels of STAT3 in whole-cell lysates and nucleus were analyzed by western blotting. GAPDH or Histone H3 was used as a control. Right: relative expression of STAT3 in whole-cell lysates and nucleus were quantified by densitometry. Results are the mean±S.E.M. (*n*=3 for each group). ^#^*P*<0.01 *versus* CTS 0 *μ*M group. (**e**) U251 cells were transfected with empty vector or STAT3C plasmid for 24 h, and then treated with CTS at the indicated concentrations for another 24 h. Cell growth was measured by MTT assay. Vehicle-treated cells were used as a control. Data are expressed as a percentage *versus* control (100%). Data are expressed as mean±S.E.M.; *n*=3 for each group. *P<0.05, **P<0.01, ***P<0.001 *versus* empty vector (EV) control; ^#^*P*<0.05, ^##^*P*<0.01. (**f**) Cell lysates from EV or STAT3C-transfected U251 cells were analyzed by western blotting with specific antibodies of STAT3 and STAT3-regulated proteins cyclin D1 and survivin. (**g** and **h**) U251 cells were treated with the indicated concentration of CTS for 24 h. The expression of proteins was analyzed by western blotting with indicated antibodies (**g**). Expression of cyclin D1, cyclin E and survivin relative to GAPDH were quantified by densitometry (**h**). Results are the mean±S.E.M. (*n*=3 for each group). *P<0.05, **P<0.01, ***P<0.001 *versus* CTS 0 *μ*M group

CTS could significantly inhibit glioma cell proliferation, however, the involved signaling pathways were not completely understood. Previously, we found CTS could inhibit STAT3 phosphorylation in U87 and T98G cells,^23^ however, the precious mechanism is unknown. Here, we confirmed that CTS significantly decreased the phosphorylation of STAT3 Tyr705 in a time- and dose-dependent manner, whereas the expression of total STAT3 protein was not changed (Figures 3a and b). We detected the activation of some upstream protein kinases of STAT3, including JAK1, 3 (Supplementary Figure S1), Akt, PTEN, GSK3*β* and ERK, as well as the other STATs (STAT1, STAT5 and STAT6). However, no changes were detected in U251 cells (Supplementary Figure S2). Immunofluorescence microscopy showed that intensity of STAT3 staining was decreased in the nucleus of cells treated with CTS (Figure 3c). Western blot also confirmed that nuclear STAT3 was reduced by CTS, but total STAT3 in cell lysate was not changed (Figure 3d). These results indicated CTS prohibited the translocation of STAT3 from the cytoplasm to nucleus through suppression its phosphorylation. Because of the importance of STAT3 in proliferation of MG cells, U251 cells were transfected with empty vector or STAT3C plasmid,^25, 26^ which is constitutively activated STAT3 but not tyrosine phosphorylated. We then explored the effect of STAT3C plasmid on CTS-mediated cell proliferation. After STAT3C plasmid transfection, the cells were treated with CTS at different concentrations for another 24 h, and then the cell growth was measured by MTT assay. Our results showed that the growth of U251 cells was inhibited by CTS in a dose-dependent manner, which was partly reversed by STAT3C overexpression (Figure 3e), indicating that the inhibitory effect of CTS on glioma cell proliferation is dependent on the suppression of STAT3 activation. As expected, cyclin D1 and survivin were increased after STAT3C overexpression (Figure 3f). The phosphorylated STAT3 is the key upstream regulators to influence on the expression of downstream proteins, including cyclin D1, survivin, which have important roles in STAT3-mediated proliferation. After U251 cells were treated with different doses of CTS (0–20 *μ*M) for 24 h, we detected the expression of these proteins. The results showed that the expression of cyclin D1 and survivin was decreased in a dose-dependent manner (Figures 3g and h).

### Inhibitory effect of CTS on p-STAT3 (Try705) was mediated by SHP-2

**Figure 4:**
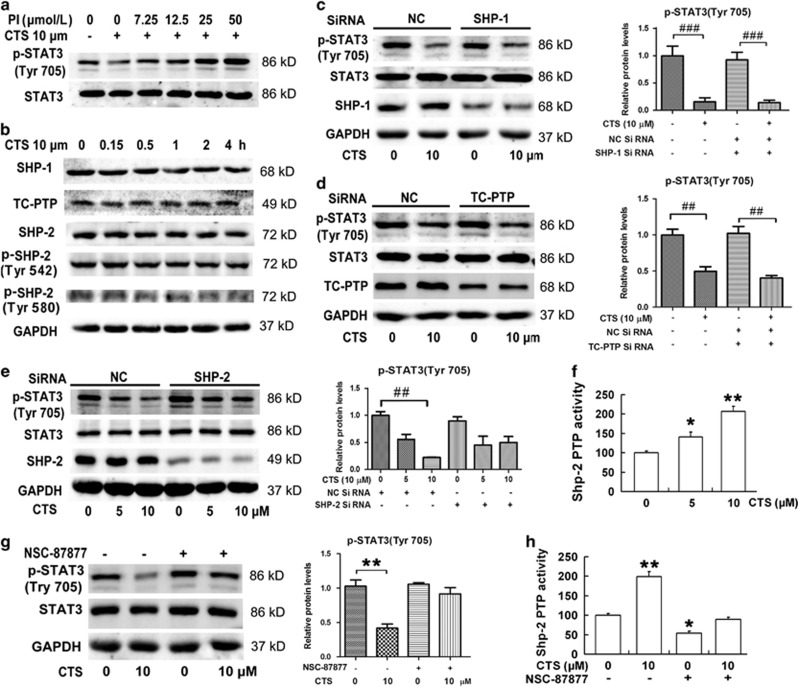
Inhibitory effect of CTS on p-STAT3 is mediated by SHP-2. (**a**) U251 cells were treated with pervanadate at indicated concentration for 1h, and with additional 10 *μ*M CTS for 2 h. Proteins were analyzed by western blotting with STAT3, p-STAT3 (Tyr705) and GAPDH antibodies. (PI: phosphoesterase inhibitors, pervanadate). (**b**) U251 cells were treated with 10 *μ*M CTS for 0, 0.15, 0.5, 1, 2, 4 h. Proteins were analyzed by western blotting with SHP-1, TC-PTP, SHP-2, p-SHP-2 (Tyr542), p-SHP-2 (Tyr580) and GAPDH antibodies. (**c**–**e**) U251 cells were transfected with specific phosphates siRNA including SHP-1 (**c**), TC-PTP (**d**), SHP-2 (**e**) or negative control (NC) for 48 h, and then treated with CTS with the indicated concentration for 120 min, followed by western blot analysis with the indicated phosphates antibodies. Expression of p-STAT3 (Tyr705) relative to STAT3 were quantified by densitometry. Results are the mean±S.E.M. (*n*=3 for each group). ^##^*P*<0.01, ^###^*P*<0.001 *versus* control. (**f**) Cell lysates from U251 cells treated with 0, 5, 10 *μ*M CTS for 2 h were subjected to SHP-2 phosphatase activity assay. Data are expressed as mean±S.E.M., *n*=3 for each group. *P<0.05; **P<0.01 *versus* CTS 0 *μ*M group. (**g** and **h**) U251 cells were pretreated with 25 *μ*M NSC-87877 for 30 min, followed by 10 *μ*M CTS treatment for 120 min. Total cell lysates were prepared and subjected to western blot analysis with indicated antibodies (**g**) or SHP-2 phosphatase activity assay (**h**). Data are expressed as mean±S.E.M., *n*=3 for each group. *P<0.05; **P<0.01, *versus* CTS 0 *μ*M group

It has been reported that PIAS3 and PTPs are negative regulatory factors of STAT3 Tyr705 phosphorylation. PIAS3 could translocate from the cytoplasm to nucleus and dephosphorylate STAT3 Tyr705.^27^ To study whether the effect of CTS on the phosphorylation of STAT3 Tyr705 was mediated by PIAS3, the expression levels of PIAS3 in the whole cell and nucleus were analyzed by western blotting. However, no significant change of PIAS3 was found (Supplementary Figure S3). To study the role of PTPs in dephosphorylation of STAT3 Tyr705 by CTS, U251 cells were treated with PTP inhibitor pervanadate (PI) at different concentrations for 1 h, and with additional 10 *μ*M CTS for 2 h, as shown in Figure 4a, CTS significantly decreased STAT3 Tyr705 phosphorylation, which was completely recovered by PI in a dose-dependent manner. This result suggests that PTPs were involved in the inhibition of STAT3 Tyr705 phosphorylation by CTS. To identify which PTP is mediated by CTS, the expression of SHP-1, TC-PTP, SHP-2, p-SHP-2 (Tyr542) and p-SHP-2 (Tyr580) protein was analyzed in U251 cells. However, there were no obvious changes in the expression of SHP-1, TC-PTP, SHP-2, p-SHP-2 (Tyr542) and p-SHP-2 (Tyr580) (Figure 4b). We next explored the influence of PTP gene silencing. The U251 cells were transfected with SHP-1, TC-PTP and SHP-2 siRNA, respectively, or NC siRNA for 48 h, and then treated with 10 *μ*M CTS for 2 h. SHP-1, TC-PTP, SHP-2 and NC siRNA sequences were shown in Supplementary Table 1. As shown in Figures 4c to e, knockdown of SHP-2 dramatically reversed the effect of CTS on the phosphorylation of STAT3 Tyr705 (Figure 4e). However, SHP-1 or TC-PTP siRNA had no effect on the reduction of phosphorylated STAT3 induced by CTS in Figures 4c and d. We further explored whether CTS could mediate SHP-2 activity. Cell lysates from U251 cells treated with 0, 5, 10 *μ*M CTS for 2 h were subjected to SHP-2 phosphatase activity assay. As shown in Figure 4f, CTS upregulated SHP-2 activity in a dose-dependent manner. The inhibitory effect of CTS on STAT3 Tyr705 phosphorylation was almost completely recovered by NSC-87877 (Figure 4g), which is the most potent SHP-1/2 inhibitor known till now.^28^ Furthermore, the PTP activity of SHP-2 increased by CTS was suppressed by NSC-87877 (Figure 4h).

### CTS inhibited intracranial tumor growth and extended survival rate of nude mice bearing intracranial U87 xenografts

**Figure 5:**
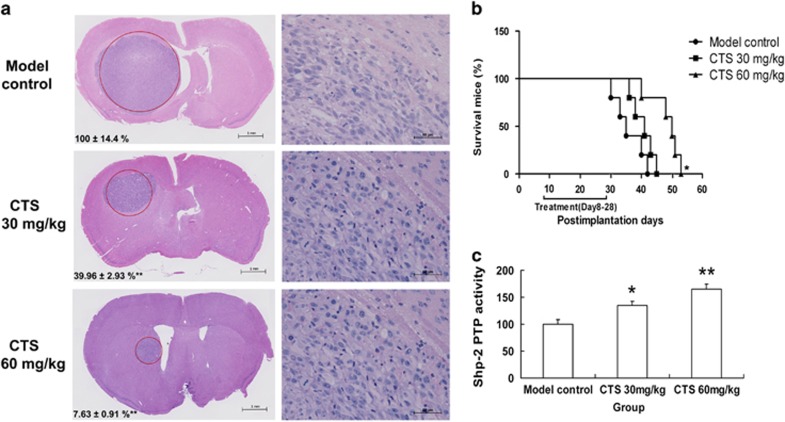
Growth inhibition of intracranial tumor by CTS treatment extended survival of nude mice bearing intracerebral U87 xenografts treated with CTS. (**a**) Growth inhibition of intracranial tumor by CTS treatment. Left: after hematoxylin–eosin staining, tumor size was determined by the areas that were measured at the maximal brain tumor dimensions in the coronal sections. The red circles indicate tumor tissue. Scale bar, 1 mm. Data were calculated by taking the tumor size of control as 100%. Data are expressed as mean±S.E.M., *n*=5 for each group. *P<0.05; **P<0.01 *versus* Model control group. Right: higher magnification in HE staining. Scale bar, 50 *μ*m. (**b**) After xenografts for 7 days, nude mice were treated with CTS from day 8 to 21 and were observed after discontinuation of therapy. Statistical significance was achieved by Cox–Mantel and Wilcoxon analyses of a Kaplan–Meier survival curve (*n*=5). (**c**) Some samples from the harvested tumor tissue on Day 28 were used in detecting tyrosine phosphatase activity of SHP-2. Data are expressed as mean±S.E.M., *n*=3 for each group. *P<0.05; **P<0.01, *versus* Model control group

U87 cells (5 × 10^5^) were injected intracerebrally. The nude mice were treated with CTS (30 mg/kg/day or 60 mg/kg/day) from day 8 to 28. On day 28, the animals were killed and their brains were harvested, fixed and sectioned. As shown in Figure 5a, there was a great discrepancy in tumor size between the CST-treated groups and the control group. The intracranial tumor growth was almost inhibited by 90% in 60 mg/kg/day CTS-treated nude mice. Also, CTS treatment could extend survival of nude mice bearing intracerebral U87 xenografts (Figure 5b). The mice treated with 60 mg/kg/day CTS lived an average of 50 days, the survival time almost extended by 43% in comparison with the control mice, which was only 35 days. To confirm the *in vivo* effect of CTS on SHP-2, we measured its tyrosine phosphatase activity in harvested tumor tissues. We found that after CTS treatment, the tyrosine phosphatase ativity of SHP-2 was increased significantly (Figure 5c).

### Immunohistochemical characterization of mouse glioma

**Figure 6:**
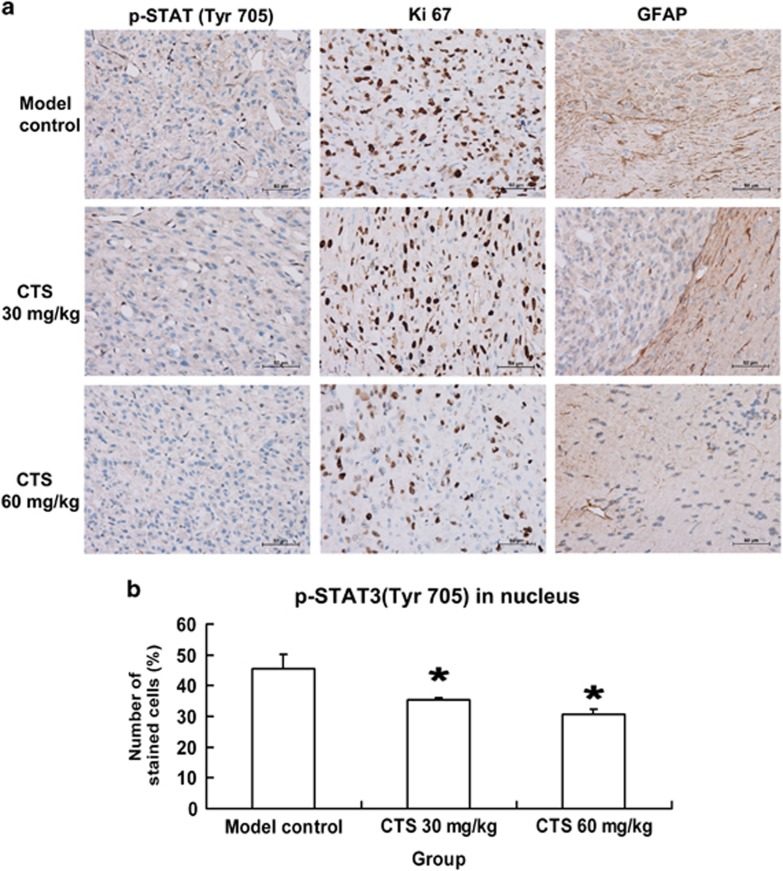
Immunohistochemical characterization of mouse glioma. (**a**) Immunohistochemical characterization of mouse glioma. Immunohistochemical staining with antibodies against p-STAT3 (Tyr705), Ki67 and glial fibrillary acidic protein (GFAP). Note the proliferation activity in brain tissue by Ki67-positive tumor cells. GFAP staining shows reactive gliosis in the brain tissue adjacent to the tumor. Scale bar, 50 *μ*m. (**b**) The levels of nuclear p-STAT3 (Tyr705) were quantified. Data are expressed as mean±S.E.M., *n*=5 for each group. *P<0.05 *versus* Model control group

To understand the effects of CTS on the characterization of mouse glioma, we then detected the expression of phosphorylated STAT3 Tyr705, Ki67 and glial fibrillary acidic protein (GFAP), an indicator for reactive gliosis in the brain tissue adjacent to the tumor by immunohistochemistry in paraffin-embedded glioma sections. The results revealed that CTS treatment reduced the intensity of p-STAT3 (Tyr705), Ki67 and GFAP staining (Figure 6a). The number of nuclear p-STAT3 (Tyr705)-positive cells was calculated and showed a dose-dependent decrease in CTS-treated groups (Figure 6b).

## Discussion

Classical therapeutic approaches for MGs include surgical resection, postoperative radiotherapy and chemotherapy (usually with temozolomide). However, since the invasive growth, incomplete surgical resection, losing insensitivity to radiotherapy and chemotherapy, it remains a high mortality and relapse rate. Therefore, more effective therapeutic approaches are urgently needed to improve the outcome of glioma treatment. CTS, as a main active component isolated from the root of *Salvia miltiorrhiza* Bunge, has been reported to possess a great antiproliferation effect on different cancer cells.^22, 29, 30, 31^ In the present study, we confirmed that CTS could significantly inhibit the proliferation of MGs cells *in vitro* and the growth of intracranial tumor *in vivo*. The survival period of nude mice bearing intracranial U87 xenografts was obviously extended. These results suggest that CTS may be a potential therapeutic agent for MGs.

Different mechanisms underlying antitumor effect of CTS have been reported in different types of tumor cells. In lung cancer cells, CTS induced autophagy via JNK signaling.^32^ In breast cancer cells, CTS inhibited breast cancer cell growth via suppressing estrogen receptor signaling.^29^ In HepG2 and MCF7 cells, CTS induced ER stress-mediated apoptosis.^33^ In colorectal cancer cells, CTS inhibited proliferation and growth via STAT3 signaling as well.^30^ Shin reported that CTS inhibited phosphorylation of STAT3 Tyr705, which was independent on the suppression of JAK2 phosphorylation.^17^ STAT3 has two phosphorylation sites, Tyr705 and Ser727. The Tyr705 site is the main phosphorylation site of STAT3. It has been reported that more than 50% p-STAT3 (Try705)-positive immunohistochemical staining in WHO grade III or IV glioma.^5^ Ser727 phosphorylation is considered a secondary event after Tyr705 phosphorylation.^34^ In our present study, we also found that the inhibitory effect of CTS on phosphorylation of STAT3 Tyr705 in MGs cells was independent on activation of JAKs. Also, our results showed that CTS had no significant effect at Ser727-phosphorylated site (Figures 3a and b). Persistent activation of STAT3 promotes its target gene (such as cycle-dependent proteins: cyclin D,^35^ survivin^36^) expression, and has a very important role in malignant growth and proliferation of MGs.^37^ In the present study, we found that CTS inhibited both cyclin D and survivin which control cell proliferation, but not cyclin E. During the regulation of cell cycle process, cyclin D1 controls the checkpoint from G1 phase to S phase;^38^ survivin controls the checkpoint from S phase to G2/M phase.^39^ Our results showed that CTS treatment increased significantly the cell percentage in G1 phase, while decreased cell percentage in G2/M phase. This result is similar as previous report,^23^ indicating that the cell cycle arrest caused by CTS treatment in MG is a whole stage process but mainly in G1 arrest.

Although the *in vitro* antitumor effect of CTS has been reported in different types of tumor cells, there have been few papers reporting *in vivo* antitumor effect of CTS,^29, 40^ and so far, its *in vivo* effect on MGs have not been reported. Compared with subcutaneous models, orthotropic xenograft model of U87 cells transplantation has more similar glioma-like growth characteristic and better stereotactic localization.^41^ We successfully established the orthotopic implantation model of human glioma cells in nude mice, which provided the tool for evaluating the therapeutic effects of CTS on MGs *in vivo*. Our results showed that CTS markedly inhibited the tumor growth with prolonged lifetime of tumor xenograft mice. CTS could also decrease the phosphorylation level of STAT3. These results were accordant with the data from cultured cells.

It has been reported that CTS has antitumor effect independent on p-JAKs, Tyk2, Src, which are the upstream kinases of STAT3 signaling.^15^ We detected the biological effect of CTS on the expression of some other upstream of STAT3 signaling, which is also MGs growth and proliferation-related, including Akt, PTEN, GSK3*β*, Erk and Chk-1.^42, 43, 44, 45^ However, no change was found in the phosphorylation of these protein kinases.

PTP SHP-2 is encoded by the *PTPN11* gene, which is also known as PTPN11 or SH-PTP2. SHP-2 was earlier reported as proto-oncogene.^46, 47, 48^ Over the past few years, a number of disease-associated SHP-2 mutations have been identified in Noonan syndrome, leukemias and other malignancies.^46, 48, 49^ Bard-Chapeau *et al.*^50^ reported that SHP-2 has a tumor-suppressor function in liver. Deletion of SHP-2 dramatically enhanced diethylnitrosamine-induced hepatocellular carcinoma development through the STAT3 pathway. The direct connection between activating mutates of PTPN11 and the disease makes SHP-2 provide an attractive target for mechanism-based therapeutics. As a PTP, SHP-2 can reduce the phosphorylation of STAT3 specifically, and is a STAT3 negative regulator.^51^ Although SHP-2 has two-way biological functions, the precise mechanisms for the pivotal role of its catalytic activity in cell signaling pathways remain to be further illustrated. Our results showed that SHP-2, but not SHP-1 and TC-PTP knockdown reversed the inhibitory effect of CTS on STAT3 phosphorylation. It indicates that SHP-2 is involved in the CTS-mediated inhibition of STAT3 signaling. It was confirmed by the result from SHP-1/2 inhibitor NSC-87877. Furthermore, we detected the tyrosine phosphatase activity of SHP-2 directly. We found that although the expression of SHP-2 protein did not change after CTS treatment, the tyrosine phosphatase activity of SHP-2 was dose-dependently increased. It is reported that SHP-2 phosphorylation at Tyr542 and Tyr580 relieves basal inhibition and stimulates SHP-2 tyrosine phosphatase activity.^52^ However, CTS did not alter either Tyr542 or Tyr580 site. So how CTS increase SHP-2 activity remains to be elucidated in our future study.

In our study, we show the *in vitro* and *in vivo* anti-MGs activity of CTS. Here, we may have a schematic summary for the mechanisms of inhibitory effect of CTS on STAT3 signaling pathway in mind, which is shown in Supplementary Figure S4. In conclusion, our results indicate that CTS may be a potential antiproliferation agent for the treatment of MGs, the mechanism may be related to the inhibition of STAT3 signaling via upregulating SHP-2 PTP activity.

## Materials and Methods

### Animals

Four- to six-week-old female BALB/c athymic nude mice were purchased from the Model Animal Research Center of Sun Yat-sen University (Guangzhou, Guangdong, China). The mice were housed and maintained in laminar air-flow cabinets under specific pathogen-free (SPF) conditions. All animal procedures were performed following the ‘Guide for the Care and Use of Laboratory Animals’ published by the National Institutes of Health (NIH) and were approved by the ethics committee of Experimental Research, Zhongshan Medical College, Sun Yat-sen University. All efforts were made to reduce the number of animals used and to minimize their suffering.

### Cell culture

C6 cells were purchased from American Type Culture Collection (ATCC, Manassas, VA, USA), and other three human MGs cell lines, U251, T98G and U87, were kindly provided by Professor Panasci (Lady Davis Institute, McGill University, Montreal, Quebec, Canada). The cells were cultured in high-glucose Dulbecco’s Modified Eagles Medium (DMEM; Invitrogen, Grand Island, New York, USA; 4.5 g/l glucose) supplemented with 10% fetal bovine serum (FBS; Invitrogen) at 37 °C under 5% (v/v) CO_2_ atmosphere.

### Chemicals, reagents and antibodies

MTT [3-(4, 5-dimethylthiazol-2-yl)-2, 5-diphenyl tetrazolium bromide] (Sigma-Aldrich, Saint Louis, MO, USA), dimethyl sulfoxide (DMSO; Sigma-Aldrich), Sodium orthovanadat (Na_3_VO_4_; Sigma-Aldrich) and CTS (C5624) (Sigma-Aldrich) used *in vitro*. CTS used *in vivo* were provided by Professor Pei-Qing Liu in Department of Pharmacology and Toxicology, School of Pharmaceutical Sciences, Sun Yat-Sen University, and was suspended in 0.5% carboxymethylcellulose sodium for intragastric administration. NSC-87877 (Tocris Bioscience, Park Ellisville, MO, USA) was used as SHP-2 inhibitor.

### SiRNA and plasmid transfection

For RNA interference, SiRNA duplexes designed against conserved targeting sequences were transfected using Lipofectamine RNAiMAX (Invitrogen, Karlsruhe, Germany) for 72 h as specified by the manufacturer. The sequence of negative control (NC), SHP-1, SHP-2 and TC-PTP SiRNA duplexes (Biomics Biotechnologies, Nantong, Jiangsu, China) were as shown in the Supplementary Table. For plasmid overexpresssion, STAT3C plasmid purchased from Addgene plasmid repository was transfected using Lipofectamine 3000 (Invitrogen) for 24 h as specified by the manufacturer.

### Detection of proliferating cells

Cell growth curve was assessed using a colorimetric MTT assay. Furthermore, cell proliferation was tested using BrdU ELISA kit according to the manufacturer's instructions (Exalpha Biologicals, Shirley, MA, USA).

### Cell migration and invasion

For detection of U251 cell migration, *in vitro* ‘scratch’ wounds were created by scraping cell monolayers with a sterile disposable rubber as described by Tamura *et al.*^53^ and U251 cells invasion experiment was evaluated using the xCELLigence System (Roche Biochemicals, Mannheim, Germany) according to the manufacturer’s protocol.

### Flow cytometry

U251 cells were treated prepared for flow cytometry detection as described previously.^23^ The percentage of cells in each phase of the cell cycle was detected by FACS Calibur Flow Cytometry (Becton Dickinson, Franklin Lakes, NJ, USA) with ModFIT using Cell cycle analysis kit (Beyotime, Suzhou, Jiangsu, China).

### Orthotopic implantation of MGs cells in nude mice

The anesthetized mice were placed in a Kopf small animal stereotaxic instrument. The syringe needle was adjusted to the bregma, then U87 cells (1 × 10^6^) suspended in 5 *μ*l of PBS were stereotactically implanted into the corpus striatum in the right cerebral hemisphere (3.5 mm deep; 1.0 mm anterior and 1.8 mm lateral to the bregma) of the nude mice, as previously described with slightly modification.^25^

### Histological analysis, immunohistochemistry and immunofluorescence

At day 28, mice were killed and the brains were removed, fixed with formaldehyde solution and sectioned routinely into 4–5 *μ*m coronal slabs, which were stained with hematoxylin and eosin (HE), followed by analysis of tumor size at the largest brain section using a light microscope as previously described with slight modification.^54^ Immunohistochemical staining was performed according to Guo's instructions.^55^ Primary antibodies were Ki67 (1 : 200; BD Transduction Laboratories, Heidelberg, Germany), GFAP (1 : 200, Cell Signaling Technology, Danvers, MA, USA), STAT3 (1 : 200, Cell Signaling Technology) and p-STAT3 (Tyr705; 1 : 100, Cell Signaling Technology).

### SHP-2 PTP activity assay

Cell or tissue lysates were subjected to immunoprecipitation (IP) with antibody against SHP-2 (BD Transduction Laboratories). The immunocomplex was pooled down by protein A/G agarose (Thermo Scientific, Schwerte, Germany) beads. SHP-2 PTP activity was measured using Tyrosine Phosphatase Assay Kit (Promega, Madison, WI, USA) as specified by its manufacturer.

### Nuclear protein isolation and western blot

Nuclear protein was extracted according to the manufacturer's instruction of the nuclear and cytoplasmic protein extraction kit (Beyotime). The protocol for western blot has been described in detail in our previous publication.^56^ Primary antibodies were used as follows: antibodies against SHP-1 and SHP-2 (Epitomics, Burlingame, CA, USA); STAT3, p-STAT3 (Tyr705), p-STAT3 (Ser727), p-p44/42 Erk1/2 (Thr202/Tyr204), cyclin D1, cyclin E1, survivin, p-SHP-2 (Tyr580), p-SHP-2 (Tyr542) and Histon H3 (Cell Signaling Technology); antibody against Ki67 (BD Bioscience); antibodies against TC-PTP, SHP-2, Mcl-1 (Proteintech, Wuhan, Hubei, China); GAPDH (Beyotime).

### Statistical analysis

All of the described experiments were performed more than three times, and the data were expressed as means±S.E.M. The statistical difference between the two experimental groups was determined using the independent-samples *t*-test, and differences among the groups were assessed by one-way ANOVA and LSD *post hoc* test. Results were analyzed using SPSS 13.0 software (IBM SPSS, Chicago, IL, USA). Statistical significance was accepted for *P*<0.05.
