# Supplementary material for: Cryptotanshinone inhibits human glioma cell proliferation in vitro and in vivo through SHP-2-dependent inhibition of STAT3 activation
Source: Cell Death Dis. 2017 May 11;8(5):e2767–. doi: 10.1038/cddis.2017.174 (PMC5520699; doi:10.1038/cddis.2017.174)
Supplement: Supplementary Information [file cddis2017174x1.pdf]

Supplementary materials

Primary antibodies used in Figure S1-3 as followes: Antibodies against p-Jak1 (Tyr1022/1023), p-Jak3 (Tyr980/981), p-STAT1, p-STAT5, p-STAT6, p-Akt (Ser473), anti-p-PTEN (Ser380), p-GSK-3β (Ser9), p-Chk1 (Ser345) and Histon H3 (Cell Signaling Technology); PIAS-3 antibody(Bioworld, St. Louis Park, MN, USA).

Supplementary figures

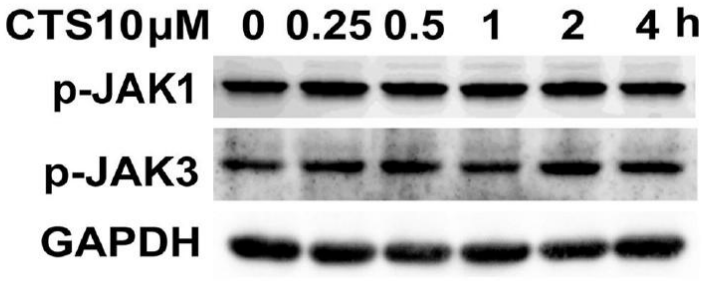

Figure S1. Effect of CTS on the phosphoryation of JAKs. U251 cells were treated with 10 µM CTS for the indicated time. Proteins were analyzed by western blotting with the indicated antibodies.

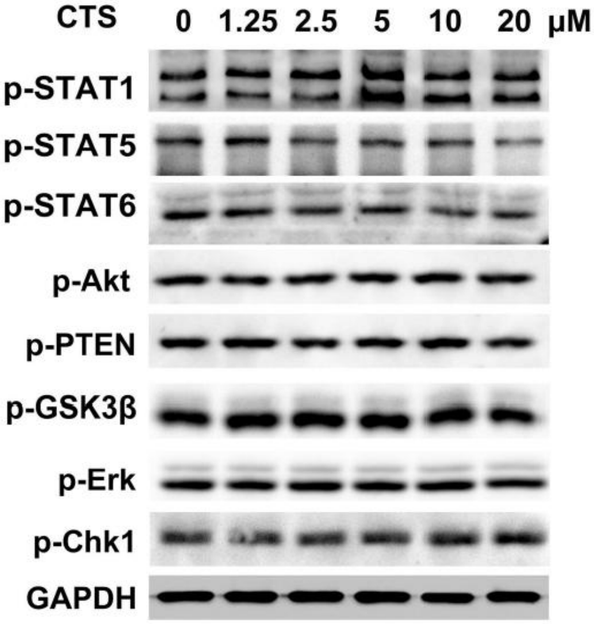

Figure S2. Effect of CTS on the expression of cell proliferation and cell cycle related proteins. U251 cells were treated with the indicated concentration of CTS for 24 hours. Proteins were analyzed by western blotting to analyze signaling molecules involved in cell proliferation.

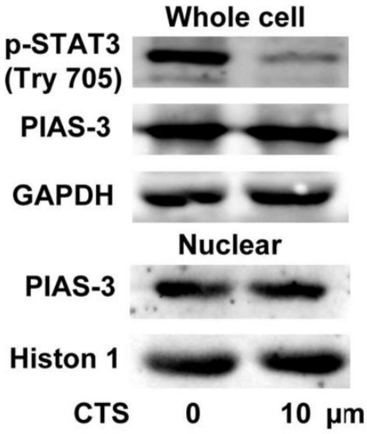

Figure S3. Effect of CTS on nuclear translocation of PIAS-3. U251 cells were treated with CTS (10 μM) for 120 min. The expression levels of PIAS-3 in the whole cell and nucleus were analyzed by western blotting. GAPDH and Histone H1 were used as the control.

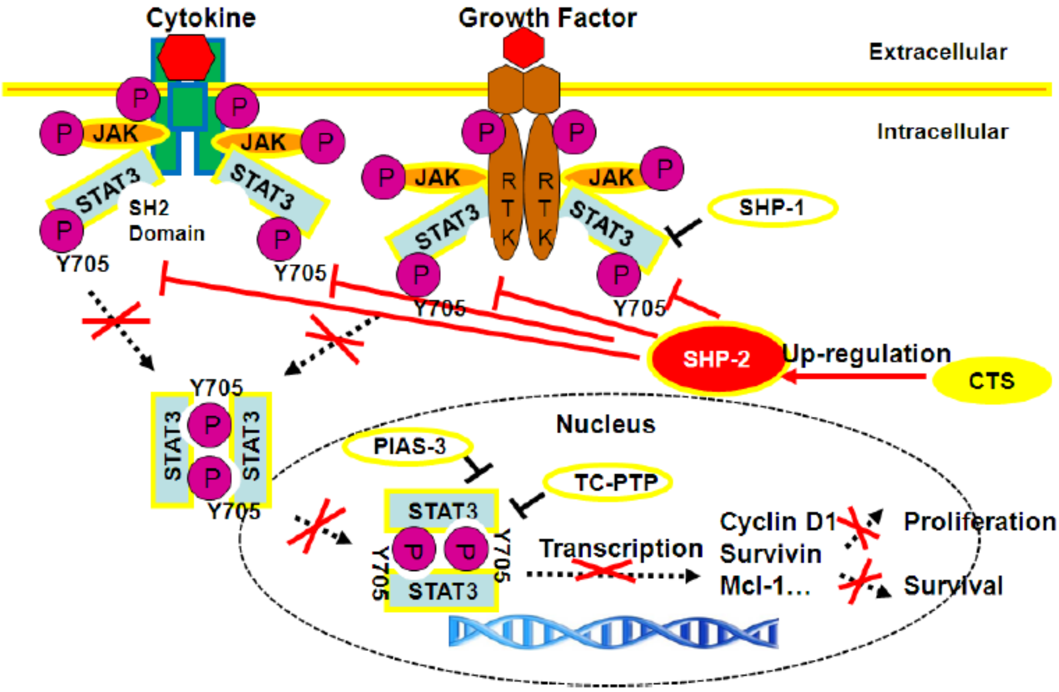

Figure S4. A schematic summary for the inhibition mechanism of CTS on STAT3 signaling pathway.
