# Supplementary material for: Cryptotanshinone inhibits human glioma cell proliferation in vitro and in vivo through SHP-2-dependent inhibition of STAT3 activation
Source: Cell Death Dis. 2017 May 11;8(5):e2767–. doi: 10.1038/cddis.2017.174 (PMC5520699; doi:10.1038/cddis.2017.174)
Supplement: Supplementary Table [file cddis2017174x2.doc]

| Gene | | Sequence |
| --- | --- | --- |
| Negative Control | Sense | 5’-UUCUCCGAACGUGUCACGUTT-3’ |
|  | Antisense | 5’-ACGUGACACGUUCGGAGAATT-3’ |
| SHP1-siRNA | Sense | 5’-GGAGAAAGGCCGGAACAAA dTdT-3’ |
|  | Antisense | 5’-dTdT CCUCUUUCCGGCCUUGUUU-3’ |
| SHP2-siRNA | Sense | 5’-GCGCACUGGUGAUGACAAA dTdT-3’ |
|  | Antisense | 5’-dTdT CGCGUGACCACUACUGUUU-3’ |
| TC-PTP- siRNA | Sense | 5’-CAAAGGAGUUACAUCUUAA dTdT-3’ |
|  | Antisense | 5’-dTdT GUUUCCUCAAUGUAGAAUU-3’ |

**Supplemental Table SiRNA sequences of NC, SHP-1, SHP-2 and TC-PTP**
